# Editorial

**Published:** 2015-09-11

**Authors:** Nikhil Marwah

**Affiliations:** Editor-in-Chief

Are conferences worth it !!!!!

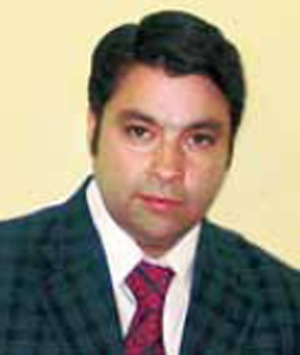

The burning question among younger dentists as well as the most seasoned clinicians and academicians is regarding the efficacy of conferences in today’s scenario. There are people who conclude that the conferences are useful for the continued growth of the subject and yet we may also find a group of people expressing their displeasure over such gatherings being just a social get-together. My choice may differ from others but the purpose of this article is to share my virtues and experiences regarding conferences in order to enlighten the younger generation of dentists with the benefits of such gatherings.

A conference is a meeting of people who ‘confer’ about a topic. Academic conference, is a formal event where researchers present results, workshops, and other activities. Since there are so many high-quality conferences and events put on each year, you really need to think about which ones you should go to and what you will get out of them by attending.

Attending a conference is a professionally rewarding experience but in the present scenario the two main functions of attending a conference are socializing with colleagues from other institutions and a trip to a possibly exotic locale. Yes, this is true with lots of people who have forgotten that the essence of conference is to hear presentations and to converse with other researchers regarding academic developments, which can always be combined with the firstly mentioned pleasures. Summarized below are some of the goals for a conference which will not only help you get the best out of it but also prepare for your success.

1. *Listening to presentations: *This will inform you of what others are doing, will inspire research ideas of your own, and will expose you to different styles of presentation.

2. *Learn: *This should be the most obvious motivation to attend conferences. As there would be many lectures going on simultaneously in the conference, be thoughtful and choosy about which sessions you commit to. Even if you do not learn something completely new, old information presented in a new light can provide a lot of inspiration.

3. *Content: *A few years ago, people went to conferences, learned lots of things and that was that. Then along came social media and everything changed. The perception amongst young dentists is that everything, which is being told in conference, is available on social media and hence the attendance has dropped. But, this is not the case; you may find the material on social media but you will not find the personal expertise of the teachers. The speakers, who specialize in these fields, have already done the sifting and selection of information from a wide variety of sources. Be observant during the conference and keep records or photos of most helpful posters and slides.

4. *Share: *Another very important aspect of yesteryears was the sharing of knowledge we acquired from these conferences wherein the students presented a summary of the proceedings to colleagues who could not attend. This not only helps you share your knowledge but also refreshes what you learned at the conference.

5. *Inspiration and motivation: *Inspiration is really everywhere when you attend an event. Conversations you have, sessions you attend, and people you meet can all help you feel inspired. The conference not only inspires but can also motivate you to try one of your ideas.

6. *Networking: *This according to me is the most important virtue of a conference and should be at the top of your to-do list in a conference. This is one of the most popular reasons people cite for attending a conference. This not only means to hang out with old friends and make new friends but also getting to talk to new people and hear what they are working on and why. At conferences, you will meet people who are interested in the same topic of research and discuss theoretical and methodological ideas. New acquaintances can be always useful in procurement of materials not available at your home department. And to top all this you always have a chance to meet you favorite teacher or your idol who is famous in the same field.

In all walks of life we encounter the experts in respective fields and always hope to be like them one day, but we all have to start somewhere. Some important things to be considered for young dentists attending their initial conferences are:

- Talk to people who have been to the conference before regarding their experiences
- Arrive a day early so that you are ready for your presentation
- Always be courteous to all
- Introduce yourself before initiating a conversation
- Approach other students to learn what they are doing and to spread the word about your own research so as to expand your circle of acquaintances
- If there is someone well-known or senior whom you want to talk, please feel free to converse as most teachers would always love to share their knowledge.
- Hang around the dining area—it is a great way to meet people;
- Plan your day well in advance and mark out all the presentations that you wish to hear
- Do not neglect the network you created at the conference—get in touch.

The focus of all individuals in the scientific and academic field is to draw a source of optimism form what is available and to use it to the fullest so as to help the younger generation to draw maximum benefits out of it and to continue the spread of knowledge. The conferences will help you to learn new vistas and dimensions in your field; get updated with the current research; build relationships with other researchers in the field; allows delegates to have issues addressed on a specific topic by recognized experts who are up-to-date with the latest developments in the field; create learning communities that bring together like-minded delegates; and the learning environment encourages delegates to exchange experiences, ideas and practices.

Always remember, as a golden rule that what is being told is not important, rather how it is being told is important and this accounts for the difference between knowing and learning.

Sometimes a little inspiration is all you need

**Nikhil Marwah**

Editor-in-Chief

